# Supplementary material for: Organization of reward and movement signals in the basal ganglia and cerebellum
Source: Nat Commun. 2024 Mar 8;15:2119. doi: 10.1038/s41467-024-45921-9 (PMC10923830; doi:10.1038/s41467-024-45921-9)
Supplement: Supplementary file 1 — Supplementary Information [file 41467_2024_45921_MOESM1_ESM.pdf]

Organization of reward and movement signals in the basal ganglia and cerebellum - Suplementary information

Noga Larry[1*], Gil Zur[1*] and Mati Joshua[1]

1. Edmond and Lily Safra Center for Brain Sciences, the Hebrew University, Jerusalem, Israel * These authors contributed equally.

Supplementary Figures

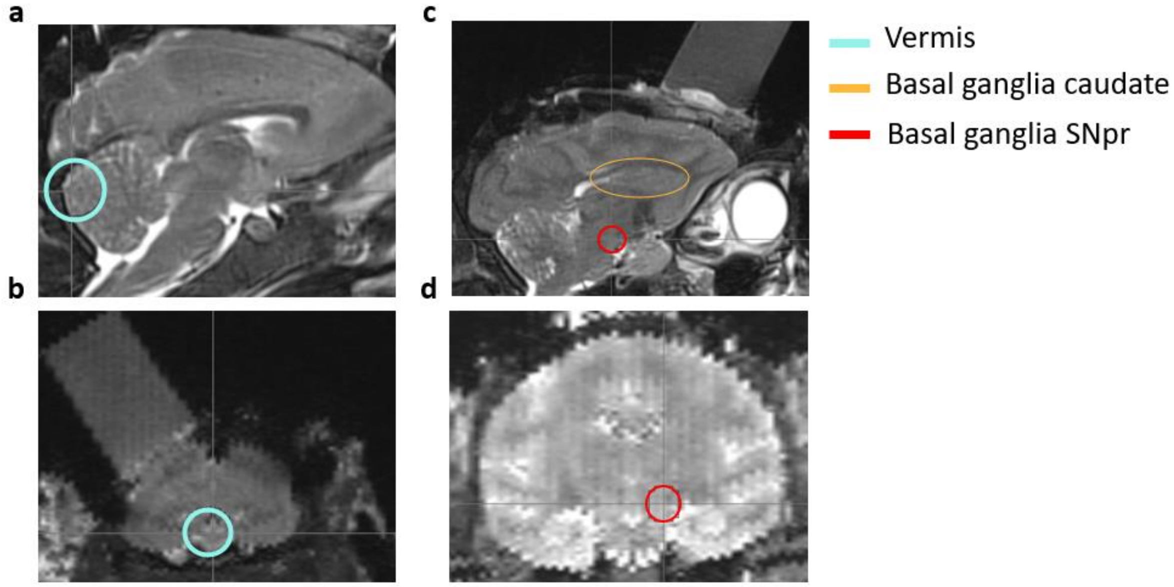

<u>**Fig. S1:**</u> **MRI sections of the recorded structures.** **a**, Mid-sagittal section showing the oculomotor vermis, marked by the cyan circle. **b**, Coronal section, 32 mm posterior to the anterior commissure, showing the recording chamber above the same area as in **a**. **c**, Sagittal section, 5 mm left of the midline, showing recording chamber above the body of the caudate, marked by the yellow ellipse, and the SNpr, marked by the red circle. **d**, Coronal section 7 mm posterior to the anterior commissure, showing the SNpr, marked by the red circle.

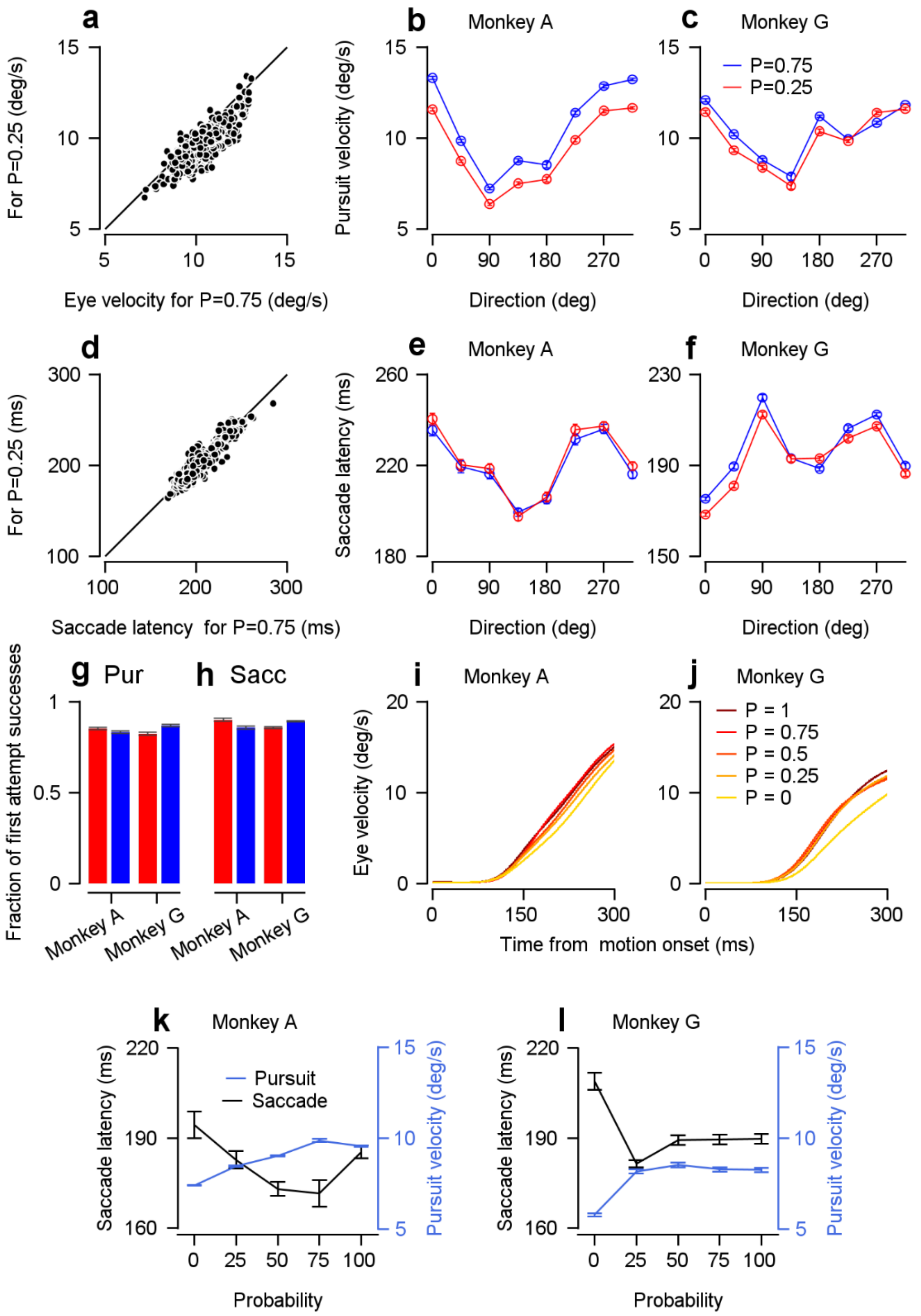

Fig. S2: Pursuit and saccade behavior in the P=0.25 and P=0.75 reward probability conditions, and sessions with five reward probability conditions. **a**, Each dot represents the average speed for an individual session 200 to 250 ms after target motion onset for the P=0.75 (horizontal) and P=0.25 (vertical) reward probability conditions (Signed-rank: $p<0.001$, $n=501$). The line shows the identity line. **b** and **c**, Pursuit velocity 200 to 250 ms after target motion onset as a function of trial direction for monkeys A (**b**) and G (**c**). **d**, Each dot represents the average saccade latency for an individual session (Signed-rank: $p<0.001$, $n=471$). **e** and **f**, Saccade latency as a function of trial direction for monkeys A (**e**) and G (**f**). **g** and **h**, Fraction of the trial that the monkey completed on the first attempt in the pursuit task (**g**) and the saccade task (**h**). **i** and **j**, Traces of average eye velocity in the first 300 ms after target motion onset in sessions with multiple probabilities on the pursuit task for monkeys A (**i**) and G (**j**). **k** and **l**, Black traces correspond to the vertical axis on the left and show the average saccade latencies

in sessions with multiple probabilities on the saccade task. Blue traces correspond to the vertical axis on the right and show the average pursuit velocity 200 to 250 ms after the target motion in sessions with multiple probabilities in the pursuit task. **k** and **l** show data from monkeys A and G.

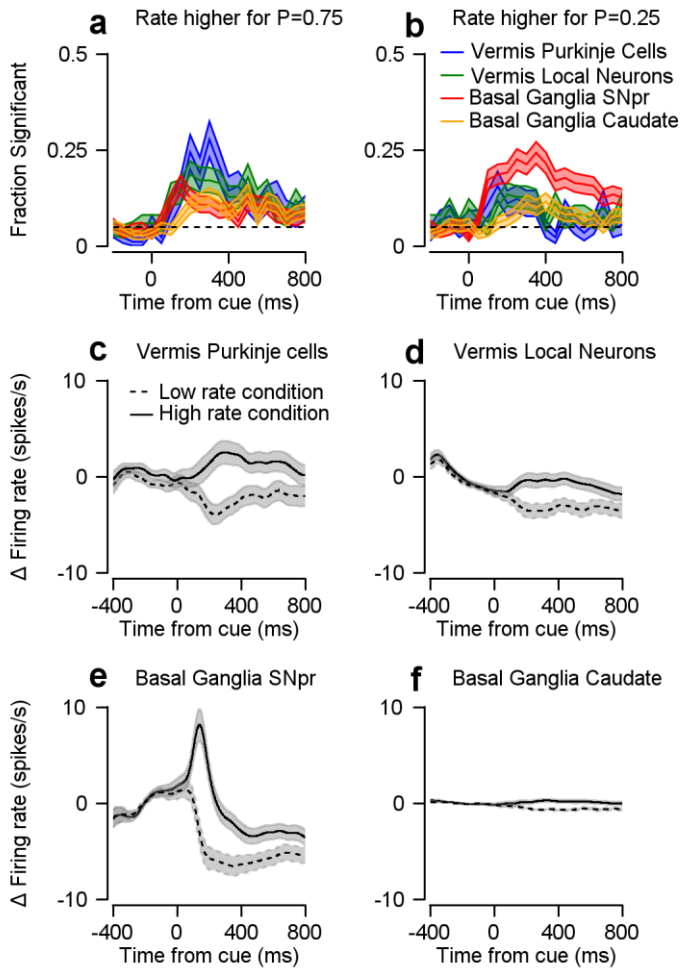

<u>Fig. S3:</u> Fraction of responsive neurons in the cue epoch and population PSTHs. a and **b**, The fraction of neurons that had a significantly higher firing rate in the P=0.75 condition (**a**) or the P=0.25 condition (**B**) in 50 ms bins (one-tailed rank-sum test). Dashed lines show the chance level. **c**-**f**, Population PSTHs for Purkinje cells (**c**), vermis local neurons (**d**), SNpr neurons (**e**), and caudate neurons (**f**). Solid traces are averaged over the reward probability condition in which the firing rate was higher in the cue epoch. Dashed lines show the average over the other condition. The baseline firing rate in the 400 ms before the cue onset was subtracted from the PSTH of each neuron.

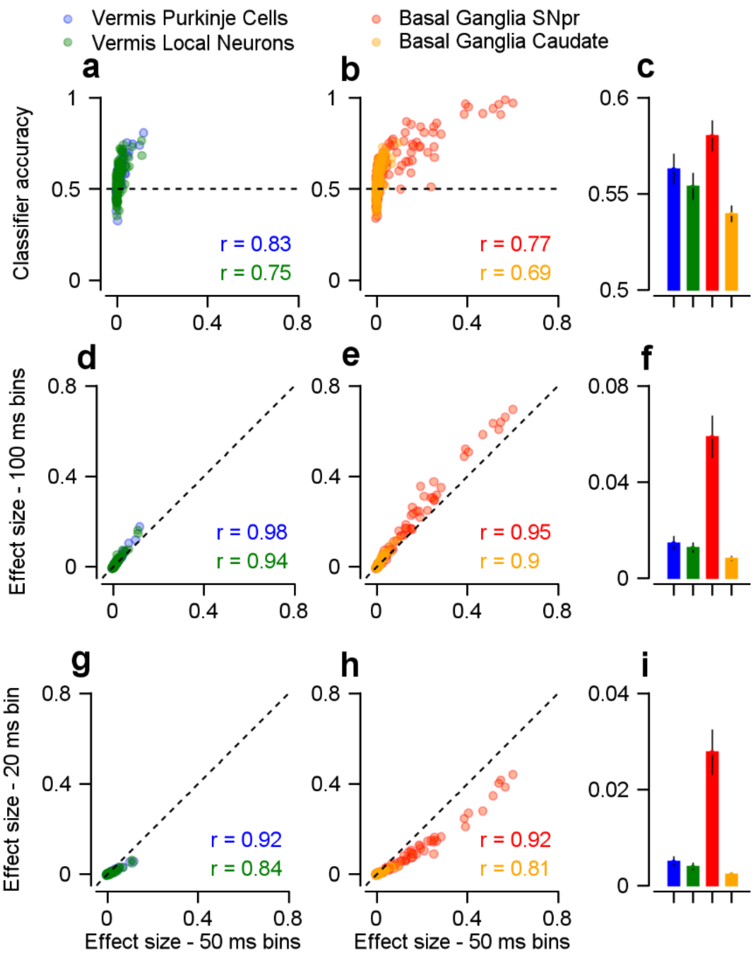

<u>Fig. S4:</u> Correlations of ω_p^2 effect sizes with the accuracy of a classifier and across different bin sizes. Each dot represents a single neuron. In all plots, the horizontal position of the dots represents the ω_p^2 reward probability effect size calculated in 50 ms bins during the cue epoch. Bar plots show the averages and standard deviations of the mean for the y-axis. The r values represent the Spearman correlations (p<0.001 for all correlations). **a**-**c**, The vertical position of each dot represents the cross-validated accuracy of a classifier that predicts the reward probability condition from the neural activity (see Methods). The dashed line represents chance accuracy. **d**-**i**, The vertical position of the dots represents the ω_p^2 reward probability effect size calculated in 100 ms (**d**-**f**) and 20 ms bins (**g**-**i**) in the cue epoch. The dashed line shows the identity line. Bar plots show the averages and standard deviations of the mean for the y-axis. **a**, **d**, and **g** show neurons from the vermis. **b**, **e**, and **h** show neurons from the basal ganglia.

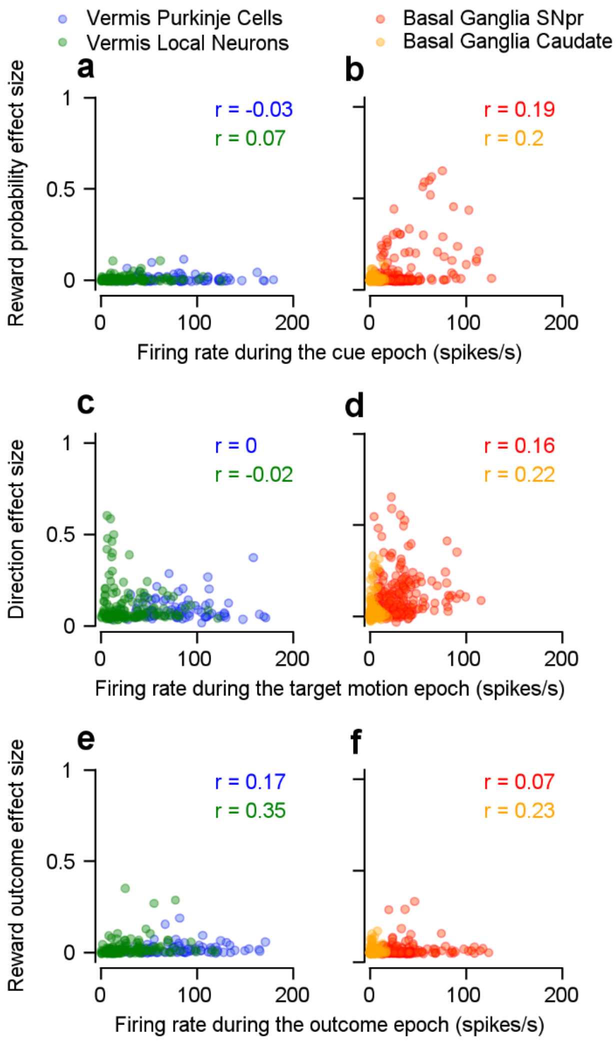

Fig. S5: Correlations between ω_p^2 and firing rate. Each dot represents the firing rate (horizontal) during a specific epoch and the effect size (vertical) for a single neuron. **a** and **b** show the reward probability effect size in the cue epoch. **c** and **d** show the direction effect size in the target motion epoch. **e** and **f** show the reward outcome effect size during the outcome epoch. **a**, **c**, and **e** show the vermis populations, and **b**, **d**, and **f** the basal ganglia. The r values represent the Spearman correlations.

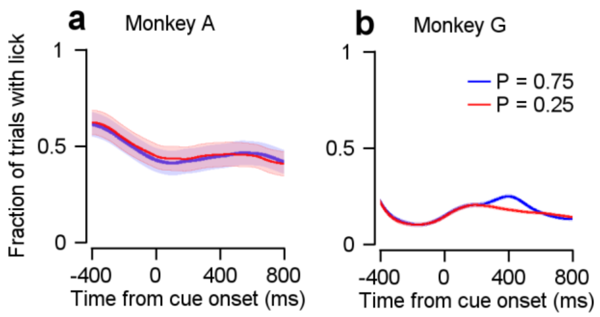

__**Fig. S6:**__ **Licking behavior in the cue epoch. a** and **b**, Fraction of trials with licks during the cue epoch in each probability condition for monkeys A (**a**) and G (**b**).

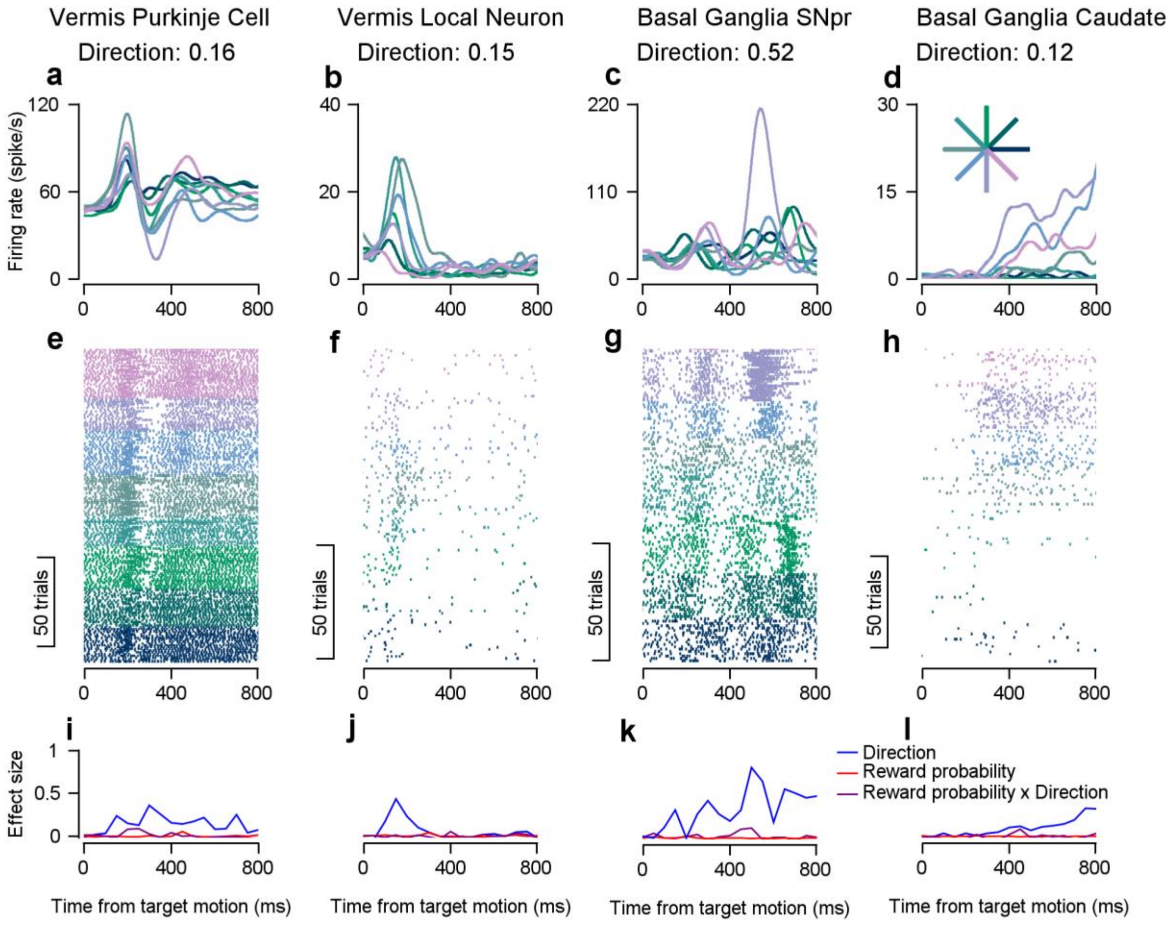

<u>Fig. S7:</u> Examples of responses to target motion in the different populations. a-d, PSTHs aligned to the onset of the target motion for the different direction conditions. **a** shows a Purkinje cell on the saccade task, **b** a vermis local neuron on the pursuit task, **c** a caudate neuron on the pursuit task, and **d** basal ganglia SNpr neuron on the pursuit task. The entire trial direction effect sizes are shown above the PSTHs. **e-h**, Raster plots for the same neurons. **i-l**, Direction (blue), reward probability (red), and reward probability x direction interaction (purple) effect sizes for the same neurons.

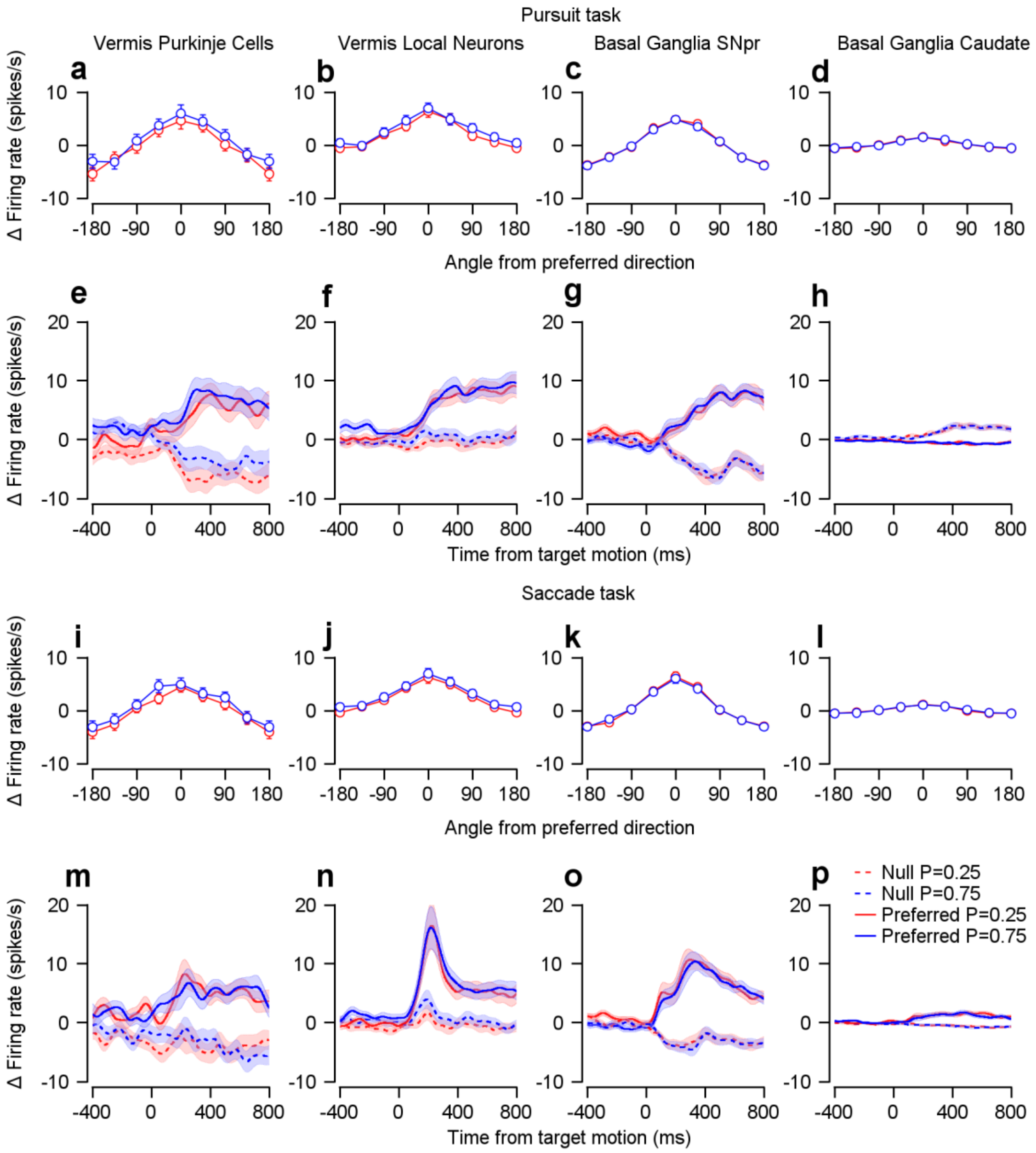

<u>Fig. S8:</u> Population PSTHs and tuning curves in the motion epoch. **a**-**d**, Population tuning curves in the pursuit task, aligned to their preferred directions. **e**-**h**, Population PSTHs in the pursuit task. **i**-**l**, Population tuning curves in the saccade task, aligned to their preferred directions. **m**-**p**, Population PSTHs in the saccade task. Different columns show different populations, indicated by the title of the column. Red and blue traces correspond to the two reward conditions. In the PSTH plots, solid lines correspond to PSTHs in the preferred direction of the neurons, and dashed lines to the null direction (180° to the preferred direction). The baseline firing rate 400 ms before the motion onset was subtracted from the PSTH and tuning curve of each neuron.

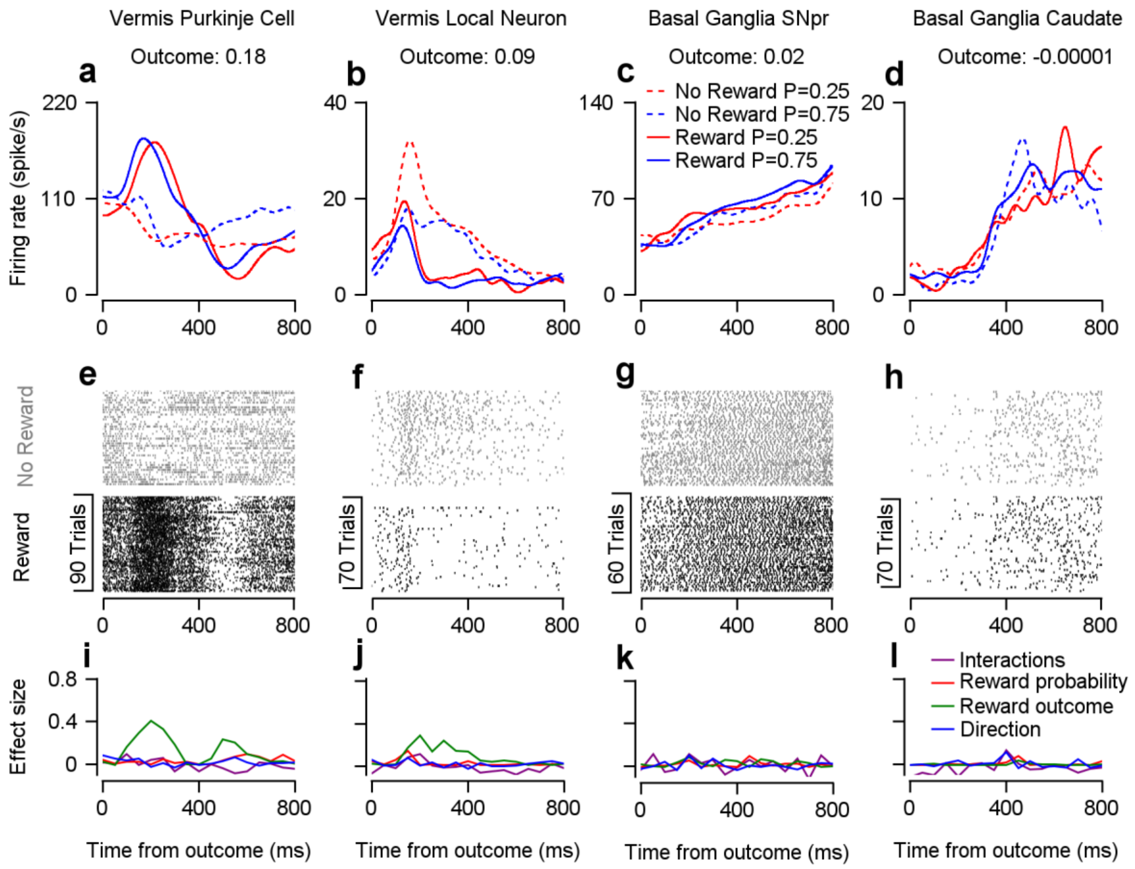

<u>Fig. S9:</u> Examples of responses in the outcome epoch in the different populations. a-d, PSTHs aligned to the end of the trial for the different reward probabilities and reward outcome conditions. **a** shows a Purkinje cell, **b** a vermis local neuron, **c** a basal ganglia SNpr neuron and **d** a caudate neuron. **e-h**, Raster plots for the same neurons. **i-l**, Direction (blue), reward probability (red), reward outcome (green), and interactions (purple) effect sizes for the same neurons.

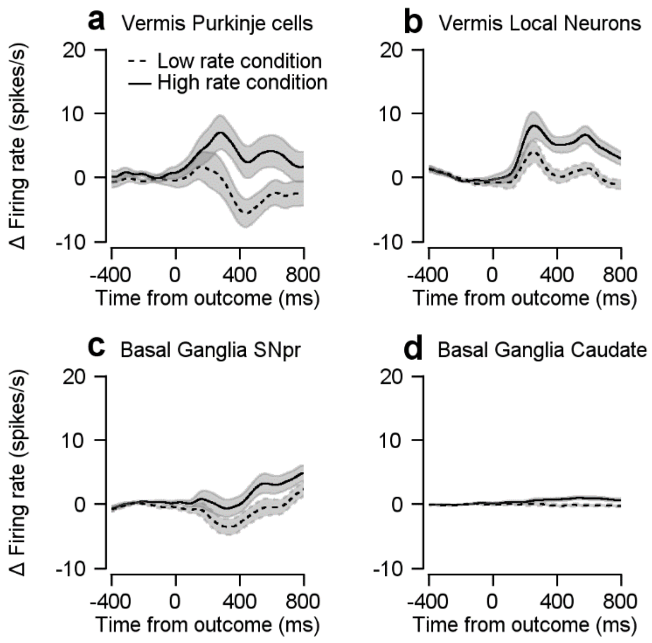

__Fig. S10:__ **Population PSTHs in the outcome epoch.** Population PSTHs for Purkinje cells (**a**), cerebellar local neurons (**b**), SNpr neurons (**c**), and caudate neurons (**d**). Solid traces are averaged over the reward outcome condition in which the firing rate was higher. Dashed lines show the average over the other condition. The baseline firing rate 400 ms before the outcome onset was subtracted from the PSTH of each neuron.

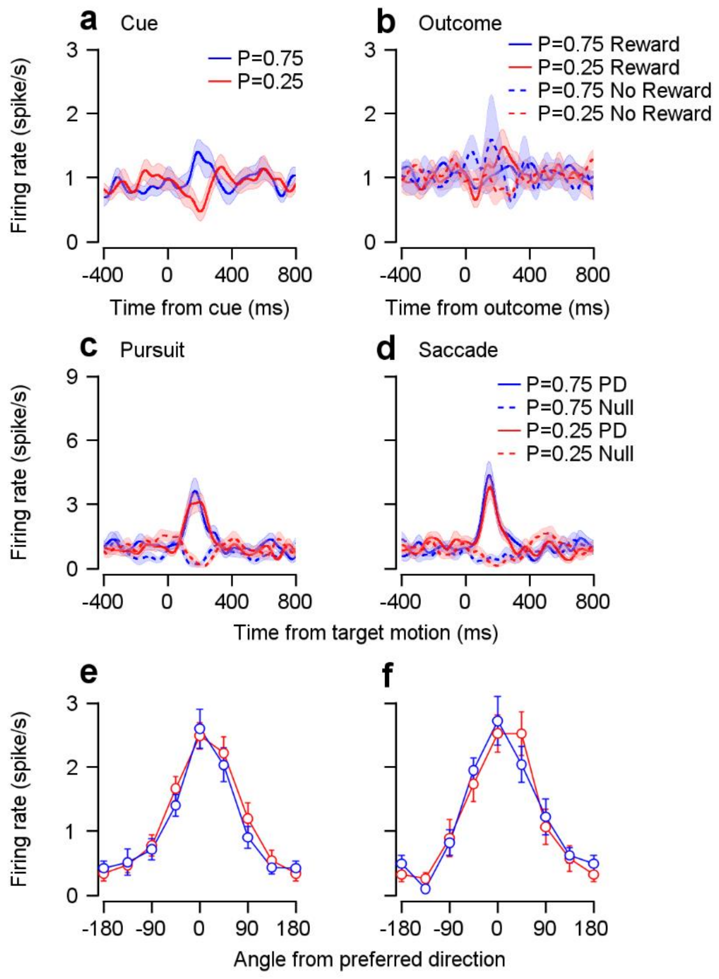

<u>Fig. S11:</u> Average complex spike responses. a-d. Population complex spike PSTHs during the different task epochs. **a**, Cue epoch, traces show the two reward probability conditions for complex spikes that significantly differentiated between reward probability conditions (ranked sum test on the 100 to 300 ms after the cue onset, 11/110 neurons). **b**, Outcome epoch, traces show different reward probability and reward outcome conditions for complex spikes that significantly differentiated between reward outcome conditions (ranked sum test on the 100 to 300 ms after the reward outcome, 16/110 neurons). **c** and **d**, Motion epoch, traces show the preferred direction (PD, solid) and the direction 180° to it (null, dashed) in the P=0.25 (red) and P=0.75 (blue) conditions in the pursuit (**c**) and saccade (**d**) tasks. **e** and **f**, Average complex spike tuning curves aligned to the preferred direction of each neuron in the pursuit (**e**) and saccade (**f**) tasks for the different reward conditions. **c**-**f** shows complex spikes that were significantly modulated by direction (Kruskal-Wallis on the 100 to 300 ms after the target motion onset, 15/73 neurons in the saccade task, 17/85 neurons in the pursuit task).

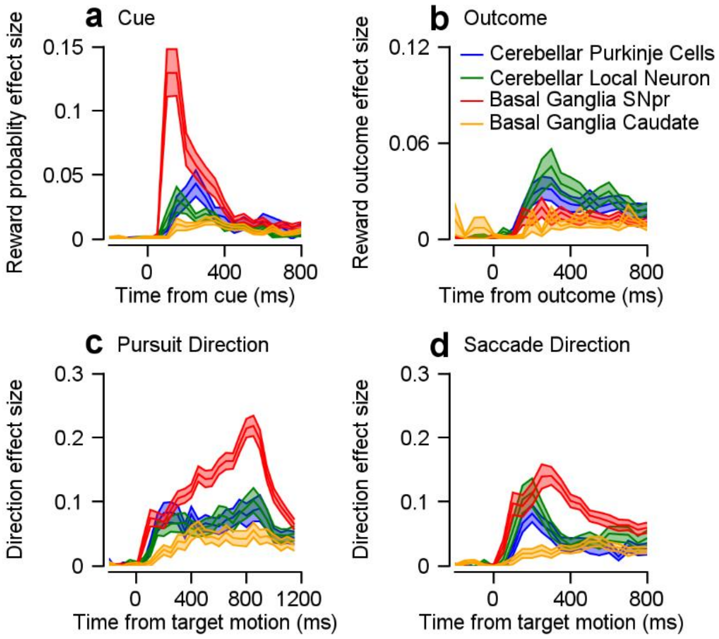

Fig. S12: Effect sizes of neurons with a significant time-varying response. a-d, This figure depicts a replication of the analysis presented in Fig. 2a (**a**), 4c (**b**), 3a (**C**), and 3b (**d**), for the subsets of neurons that had a significant time-varying response in the corresponding epoch (see Methods). Repeating the statistical tests did not alter the conclusions reported in the main text. Test for **a**: Permutation Welch's ANOVA test: $p < 0.001$, Permutation Welch's t-test: $p_{SNpr\text{-}caudate}$, $p_{SNpr\text{-}Purkinje}$, $p_{SNpr\text{-}local} < 0.001$. Test for **b:** Permutation Welch's ANOVA test: $p < 0.001$, Permutation Welch's t-test: $p_{SNpr\text{-}vermis}$, $p_{caudate\text{-}vermis} < 0.001$, $p_{Purkinje\text{-}local} = 0.33$. Test for **c**: Permutation Welch's ANOVA test: $p < 0.001$, Permutation Welch's t-test: $p_{SNpr\text{-}caudate}$, $p_{SNpr\text{-}Purkinje} < 0.001$. Test for **d**: Permutation Welch's ANOVA test: $p < 0.001$, Permutation Welch's t-test: $p_{SNpr\text{-}caudate}$, $p_{SNpr\text{-}Purkinje} < 0.001$, $p_{SNpr\text{-}local} = 0.41$).

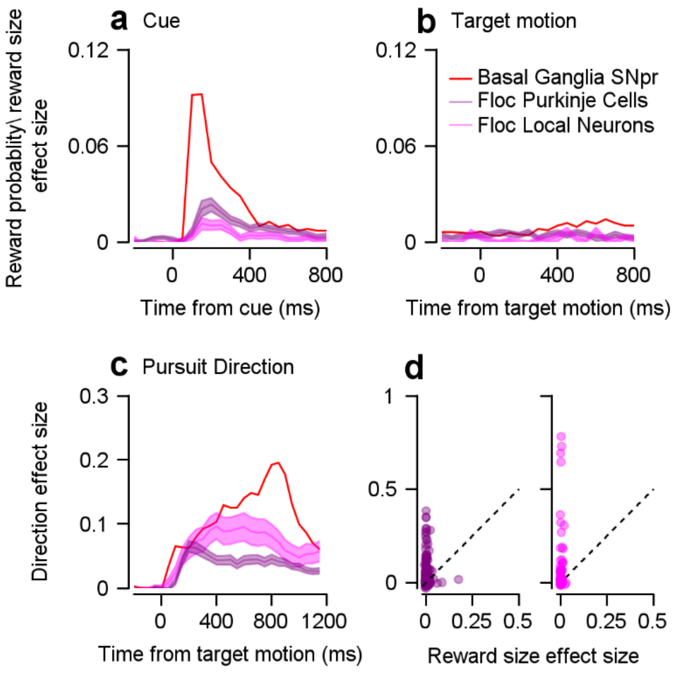

<u>Figure S13:</u> Effect sizes of neurons in the floccular complex of the cerebellum and neighboring areas during pursuit. Effect sizes were calculated for neurons collected from two other monkeys in a previous study[1,2]. The monkeys performed a similar pursuit task in which the cue indicated the future reward size. Pink traces represent Purkinje cells in the floccular complex and purple traces represent local neurons. Red traces are the average effect size of the SNpr shown previously. **a** and **b**, Average reward size or reward probability effect size in the cue (**a**; Permutation Welch's t-test: $p_{SNpr\text{-}Purkinje}$, $p_{SNpr\text{-}local}<0.001$) and target motion (**b**; Permutation Welch's t-test: $p_{SNpr\text{-}Purkinje}$, $p_{SNpr\text{-}local}<0.001$). **c**, Average direction effect size in the target motion epoch (Permutation Welch's t-test: $p_{SNpr\text{-}Purkinje}<0.001$, $p_{SNpr\text{-}local}=0.05$). **d**, Each dot represents a single neuron's reward probability (horizontal) and direction (vertical) effect sizes in the target motion epoch (Bootstrap t-test: $p_{Purkinje}$, $p_{local}<0.01$).

Supplementary Tables

	Pursuit task	Saccade task	At least one task
Caudate	86/75	62/107	109/123
SNpr	62/104	38/112	66/165
Local cerebellar neurons	61/42	37/50	72/62
Purkinje cell simple spikes	39/32	29/36	47/43
Purkinje cell complex spikes	56/29	35/38	64/46
Simple and complex spike pairs	36/23	27/26	38/29

Table S1: Sample sizes. Number of neurons recorded from each monkey on each task. The left and right values show the number of neurons for Monkeys A and G.

	Cue	motion: pursuit	motion: saccade	Outcome
Purkinje cell simple spikes	48 (0.53)	49 (0.69)	45 (0.69)	86 (0.95)
Local cerebellar neurons	78 (0.58)	70 (0.67)	66 (0.75)	118 (0.88)
SNpr	162 (0.7)	146 (0.87)	123 (0.82)	213 (0.92)
Caudate	92 (0.39)	71 (0.44)	104 (0.61)	171 (0.73)

Table S2: **Number of neurons with a significantly time-varying response.** The number and fraction (in parenthesis) of neurons that were included in the analysis presented in Fig. S10.
