# Supplementary material for: Organization of reward and movement signals in the basal ganglia and cerebellum
Source: Nat Commun. 2024 Mar 8;15:2119. doi: 10.1038/s41467-024-45921-9 (PMC10923830; doi:10.1038/s41467-024-45921-9)
Supplement: Supplementary file 4 — Source Data [file 41467_2024_45921_MOESM4_ESM.zip › Accessing data in fyp files.pdf]

Organization of reward and movement signals in the basal ganglia and cerebellum

Noga Larry[1*], Gil Zur[1*] and Mati Joshua[1]

1. Edmond and Lily Safra Center for Brain Sciences, the Hebrew University, Jerusalem, Israel
   * These authors contributed equally.

Accessing data in fyp files

The fyp files can be opened using Figure Composer, a free software that can be downloaded here. The fyp files contain the figures in the paper and allow to access the values of individual data points.

After opening the relevant figure file, click on the data you are interested in.

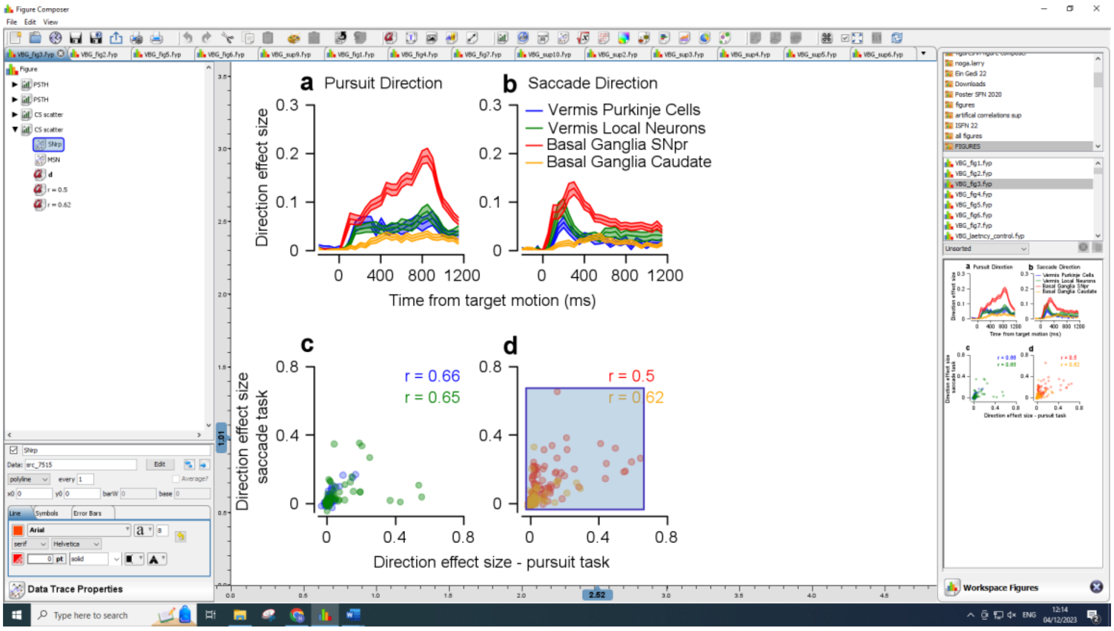

Then click on the "edit" button.

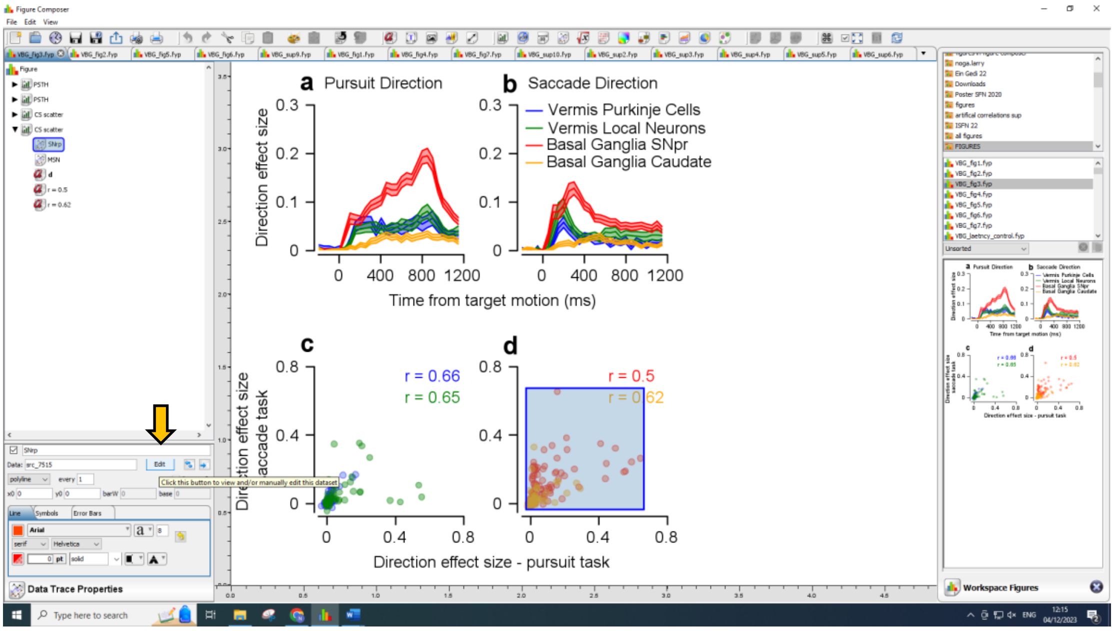

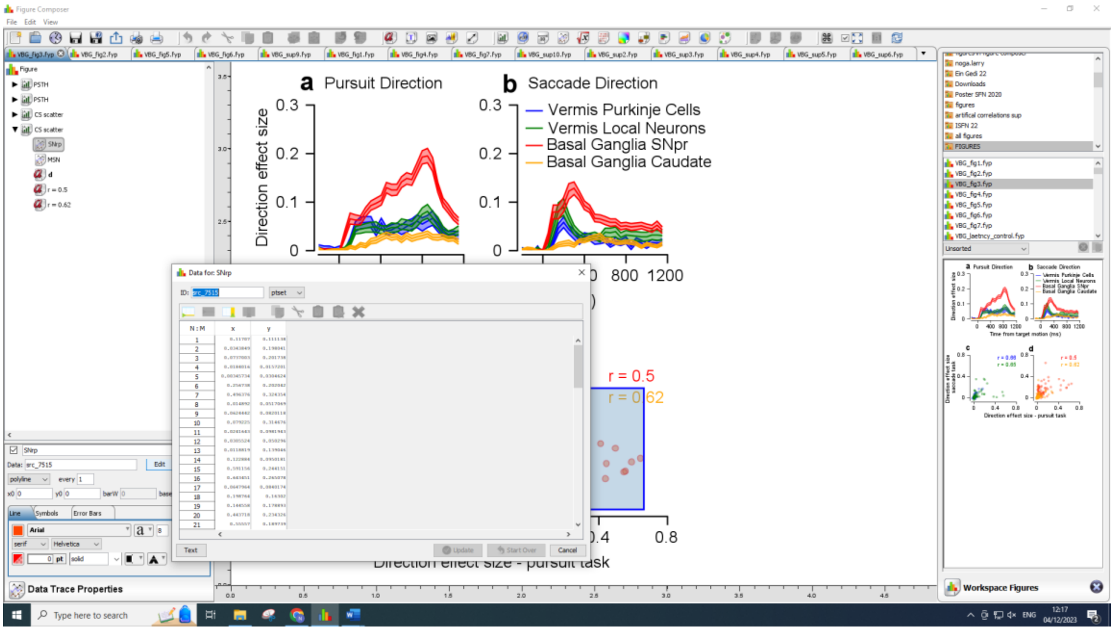

Each row corresponds to a data point on the graph and shows the horizontal and vertical values.
